# Supplementary material for: Protein Profiling of Breast Carcinomas Reveals Expression of Immune-Suppressive Factors and Signatures Relevant for Patient Outcome
Source: Cancers (Basel). 2022 Sep 19;14(18):4542. doi: 10.3390/cancers14184542 (PMC9496820; doi:10.3390/cancers14184542)
Supplement: Supplementary file 1 [file cancers-14-04542-s001.zip › cancers-1917194-supplementary File S1.pdf]

Supporting Material for Figure 3C: Shown are DigiWest data for 3 representative hot and cold patients each. Data is presented as median fluorescence intensities plotted against the molecular weight of the respective protein fraction, resulting raw signals after integration and as resulting digital reconstructed greyscale maps. For more information please see Treindl et al., 2016 (https://doi.org/10.1038/ncomms12852).

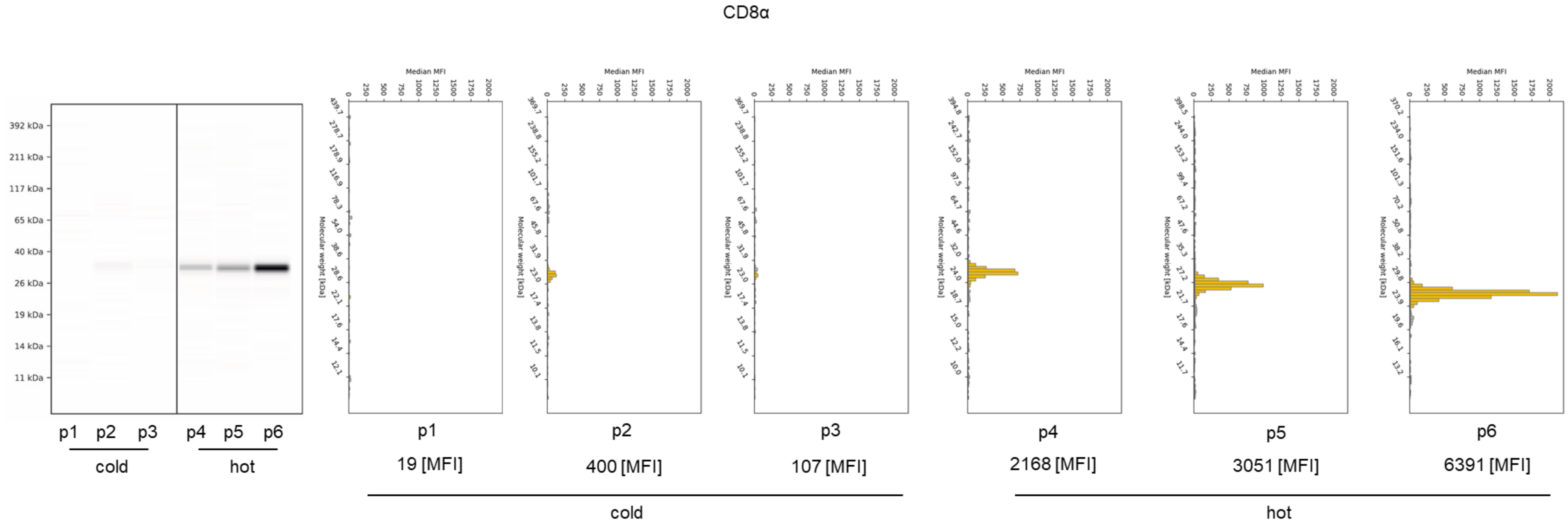

CD11c

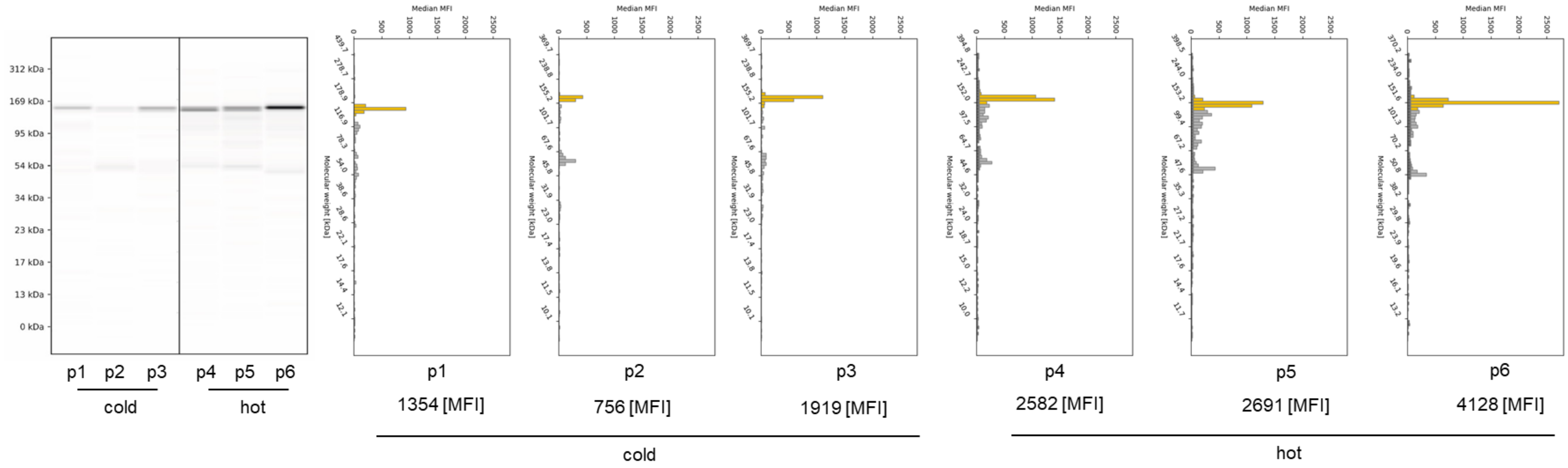
312 kDa
169 kDa
95 kDa
54 kDa
34 kDa
23 kDa
17 kDa
13 kDa
0 kDa
p1 p2 p3 p4 p5 p6
cold
hot
Median MFI
Molecular weight [kDa]
p1
1354 [MFI]
p2
756 [MFI]
p3
1919 [MFI]
p4
2582 [MFI]
p5
2691 [MFI]
p6
4128 [MFI]
cold
hot

CD16

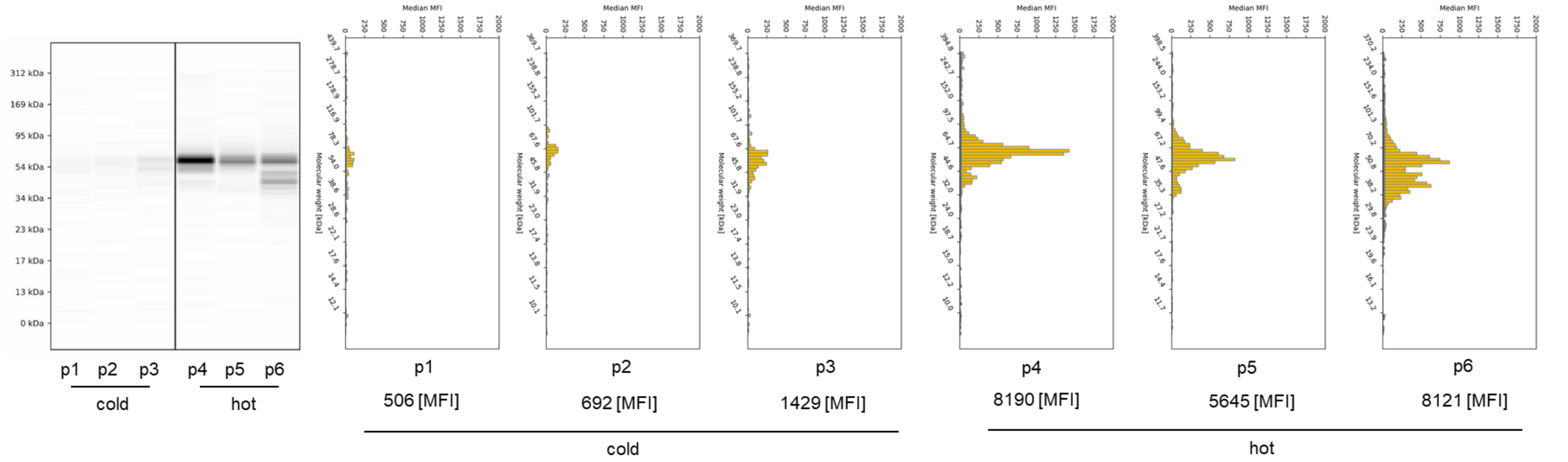
312 kDa
169 kDa
95 kDa
54 kDa
34 kDa
23 kDa
17 kDa
13 kDa
0 kDa
p1 p2 p3
cold
p4 p5 p6
hot
Median MFI
Molecular weight [kDa]
p1
506 [MFI]
p2
692 [MFI]
p3
1429 [MFI]
p4
8190 [MFI]
p5
5645 [MFI]
p6
8121 [MFI]
cold
hot

CD68

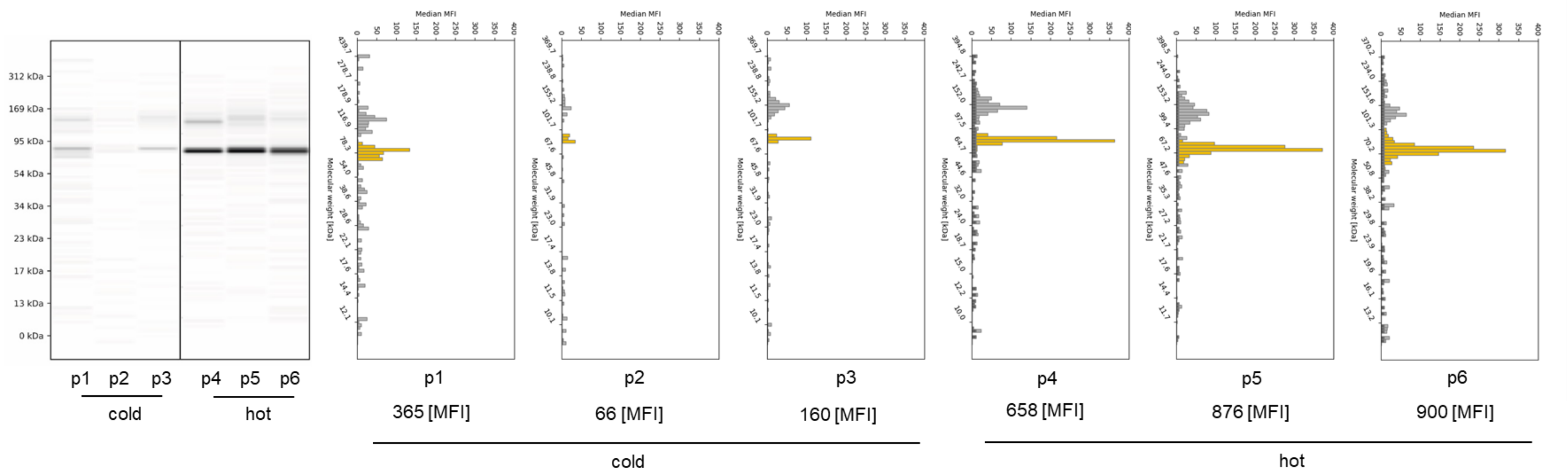

312 kDa
169 kDa
95 kDa
54 kDa
34 kDa
23 kDa
17 kDa
13 kDa
0 kDa
p1 p2 p3
cold
p4 p5 p6
hot
Median MFI
Molecular weight [kDa]
p1
365 [MFI]
p2
66 [MFI]
p3
160 [MFI]
p4
658 [MFI]
p5
876 [MFI]
p6
900 [MFI]
cold
hot

Supporting Material for Figure 4C: Shown are DigiWest data for 2 representative hot and cold patients. Data is presented as median fluorescence intensities plotted against the molecular weight of the respective protein fraction, resulting raw signals after integration and as resulting digital reconstructed greyscale maps. For more information please see Treindl et al., 2016 (https://doi.org/10.1038/ncomms12852).

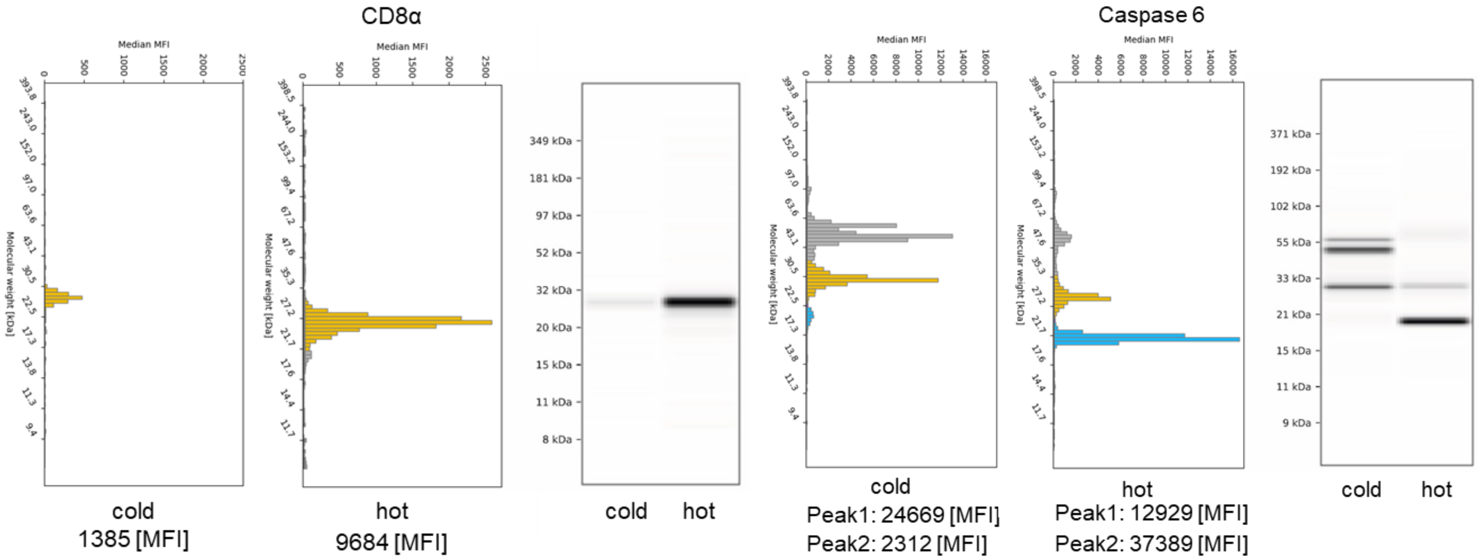

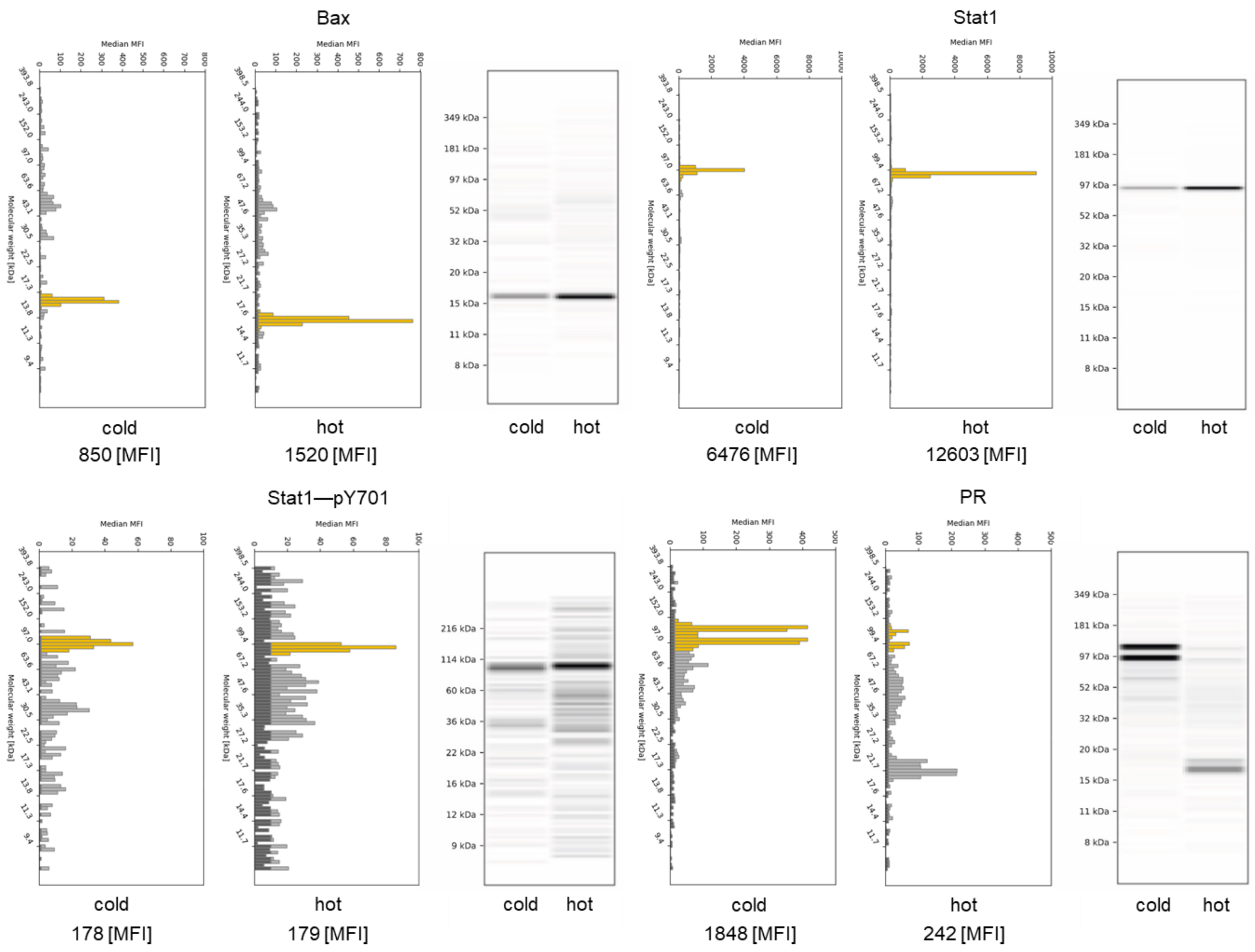
Bax
Stat1
cold
850 [MFI]
hot
1520 [MFI]
cold
6476 [MFI]
hot
12603 [MFI]
Stat1—pY701
PR
cold
178 [MFI]
hot
179 [MFI]
cold
1848 [MFI]
hot
242 [MFI]

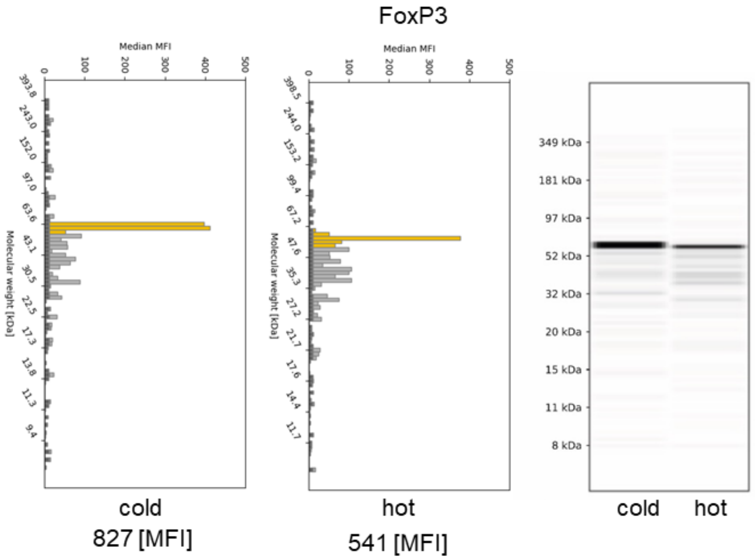
FoxP3
Median MFI
Molecular weight [kDa]
cold
827 [MFI]
hot
541 [MFI]
cold
hot

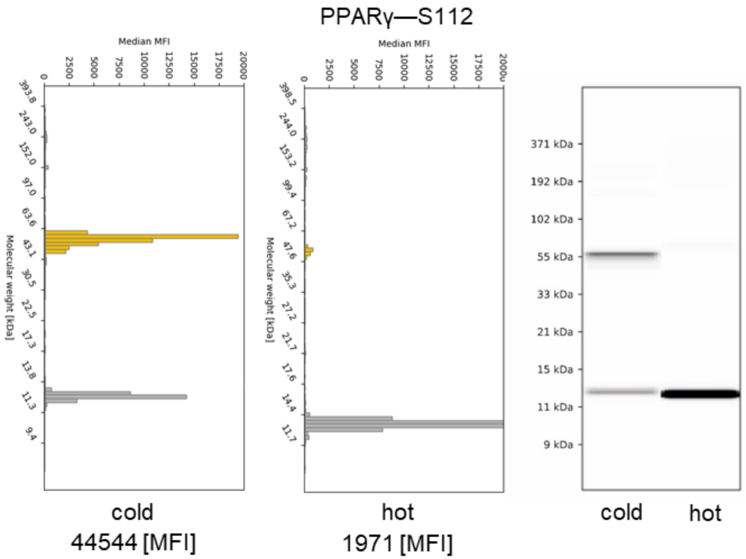
PPARγ—S112
Median MFI
Molecular weight [kDa]
cold
44544 [MFI]
hot
1971 [MFI]
cold
hot

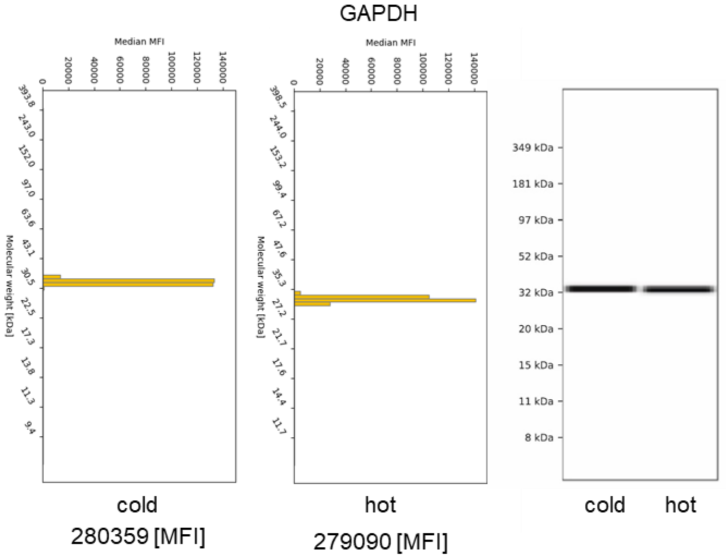
GAPDH
Median MFI
Molecular weight [kDa]
cold
280359 [MFI]
hot
279090 [MFI]
cold
hot
