# Supplementary material for: Protein Profiling of Breast Carcinomas Reveals Expression of Immune-Suppressive Factors and Signatures Relevant for Patient Outcome
Source: Cancers (Basel). 2022 Sep 19;14(18):4542. doi: 10.3390/cancers14184542 (PMC9496820; doi:10.3390/cancers14184542)
Supplement: Supplementary file 1 [file cancers-14-04542-s001.zip › cancers-1917194-supplementary.pdf]

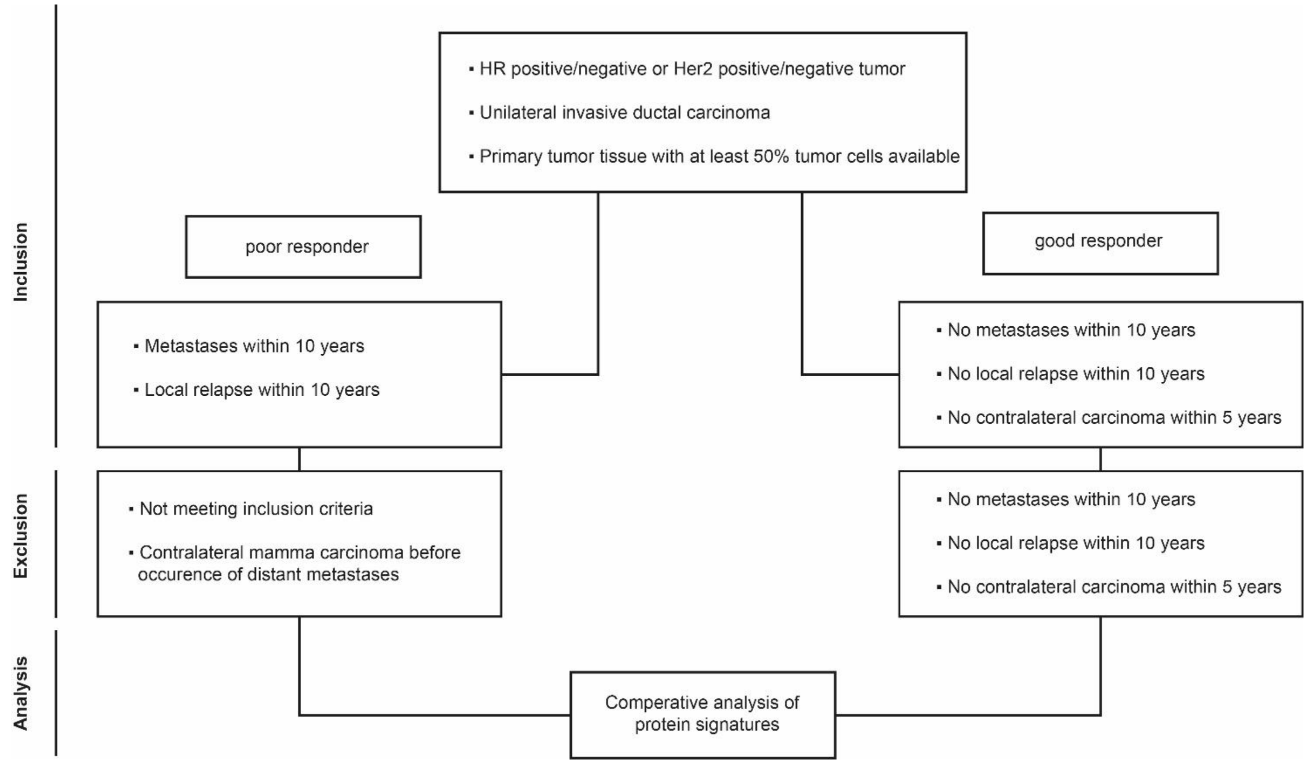

Supplementary Figure S1. Study flowchart.

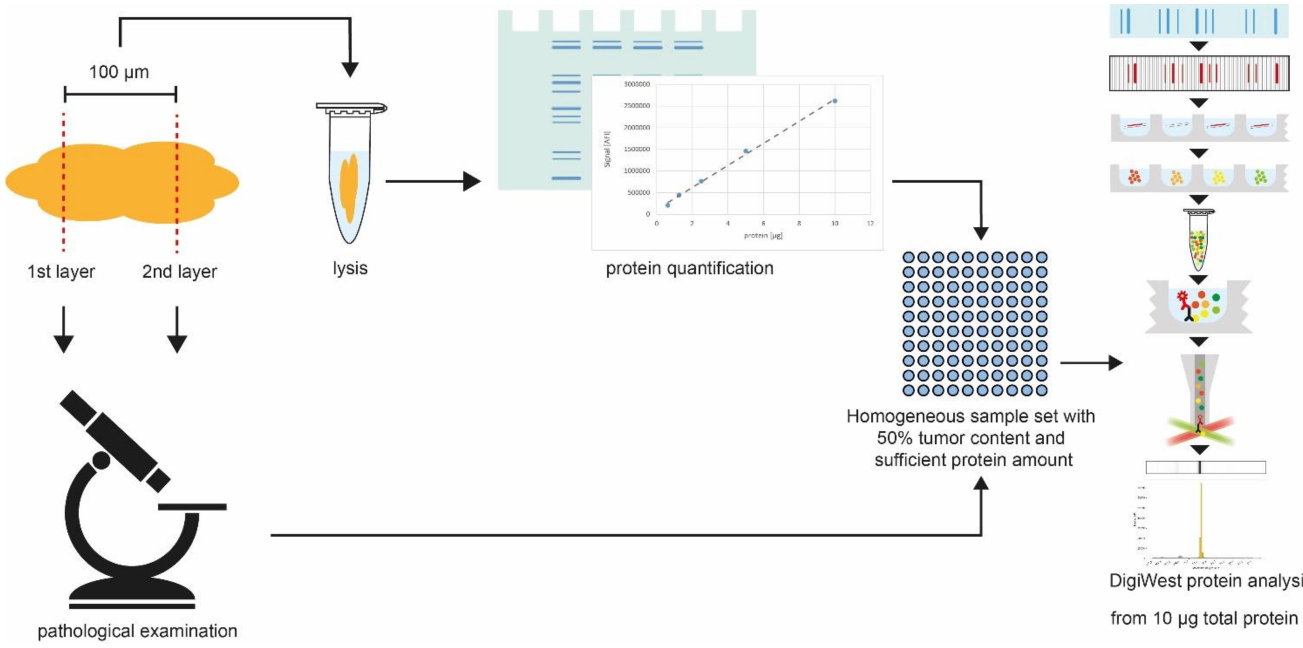

Supplementary Figure S2. Schematic depiction of study workflow. Layered cuts of tumor biopsies were generated, and intermediate tissue (100 µm) was collected. A pathologist assessed tumor content in first and second layer. Collected sample material was lysed and protein quantification was performed. Samples with tumor content of ≥50 % tumor content and sufficient protein amount were selected for DigiWest analysis.

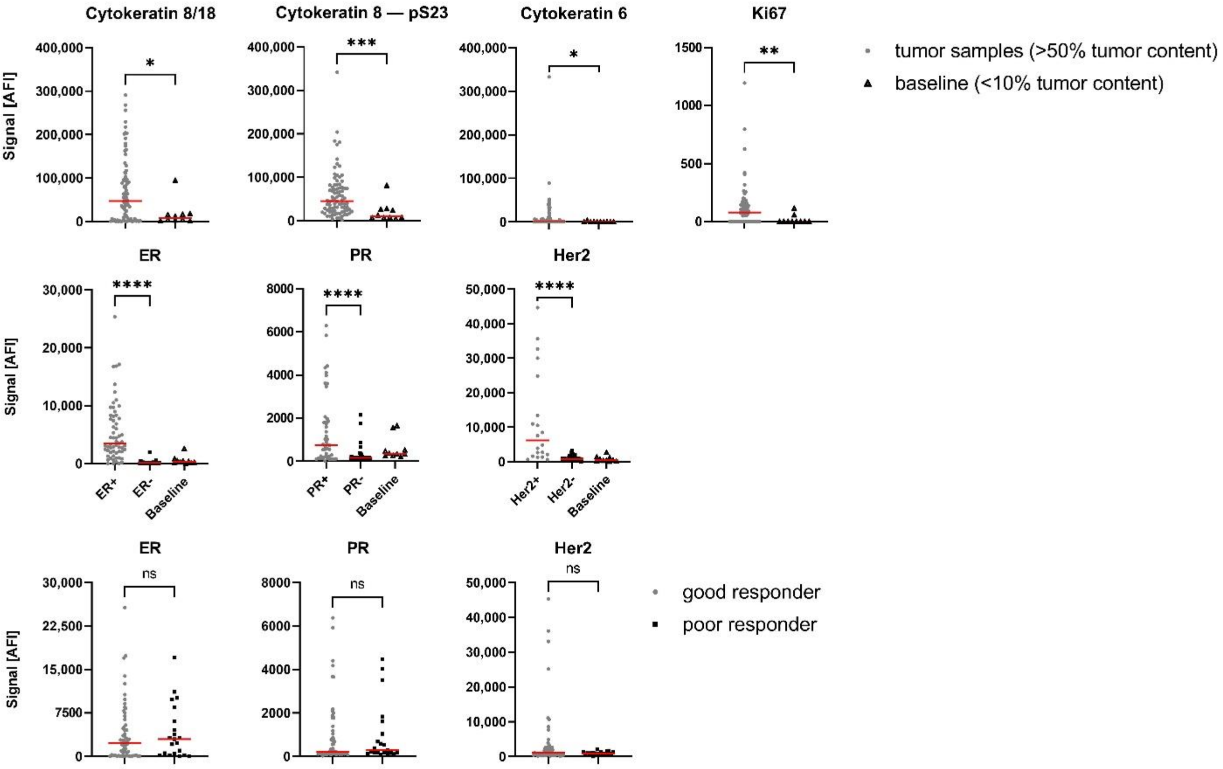

Supplementary Figure S3. Tumor marker and receptor expression in baseline samples versus tumor samples. (A) Cytokeratin 8/18, Cytokeratin 8 – pS23, Cytokeratin 6 and Ki67 expression as scatter plots in samples with ≥ 50 % tumor content (Tumor samples, n=84) and ≤10% tumor content (baseline, n=10). (B) Scatter plots showing protein expression of ER, PR and Her2 in respective receptor-positive or negative and baseline subgroup (ER+ n=60; ER- n=24; PR+ n=46; PR- n=38; Her2+ n=20; Her2- n=63) as well as (C) in good (n=58) and poor responders (n=21). In A, B, C Mann-Whitney-U test, ****P<0.0001; ***P<0.001; **P<0.01; *P<0.05; ns indicates no significant difference.

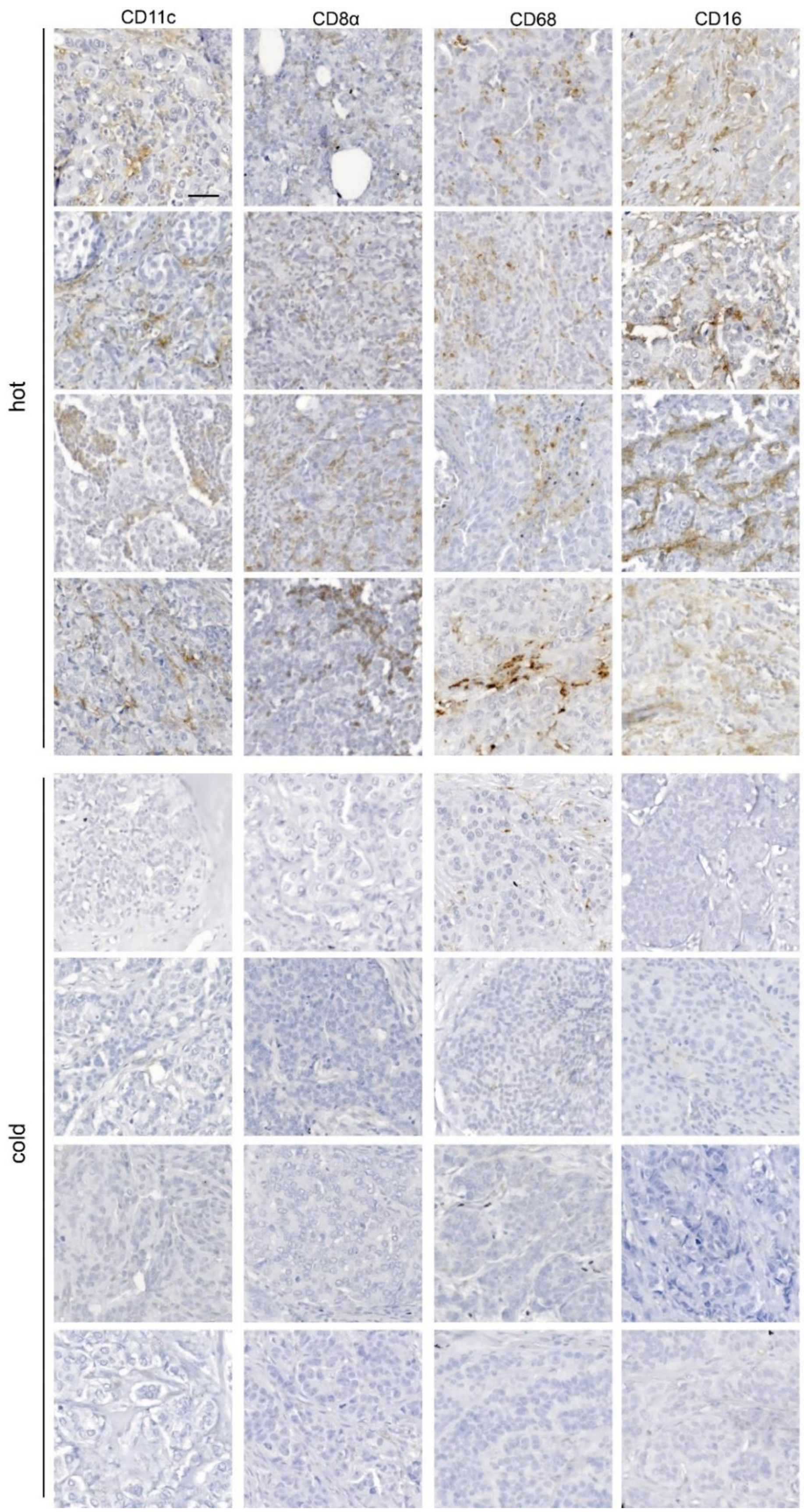

Supplementary Figure S4. Overview of IHC staining of immune cells. Representative images of CD11c, CD8, CD68 and CD16 immuno-histochemical staining in hot and cold samples. n=4. Scale bar, 50 μm.

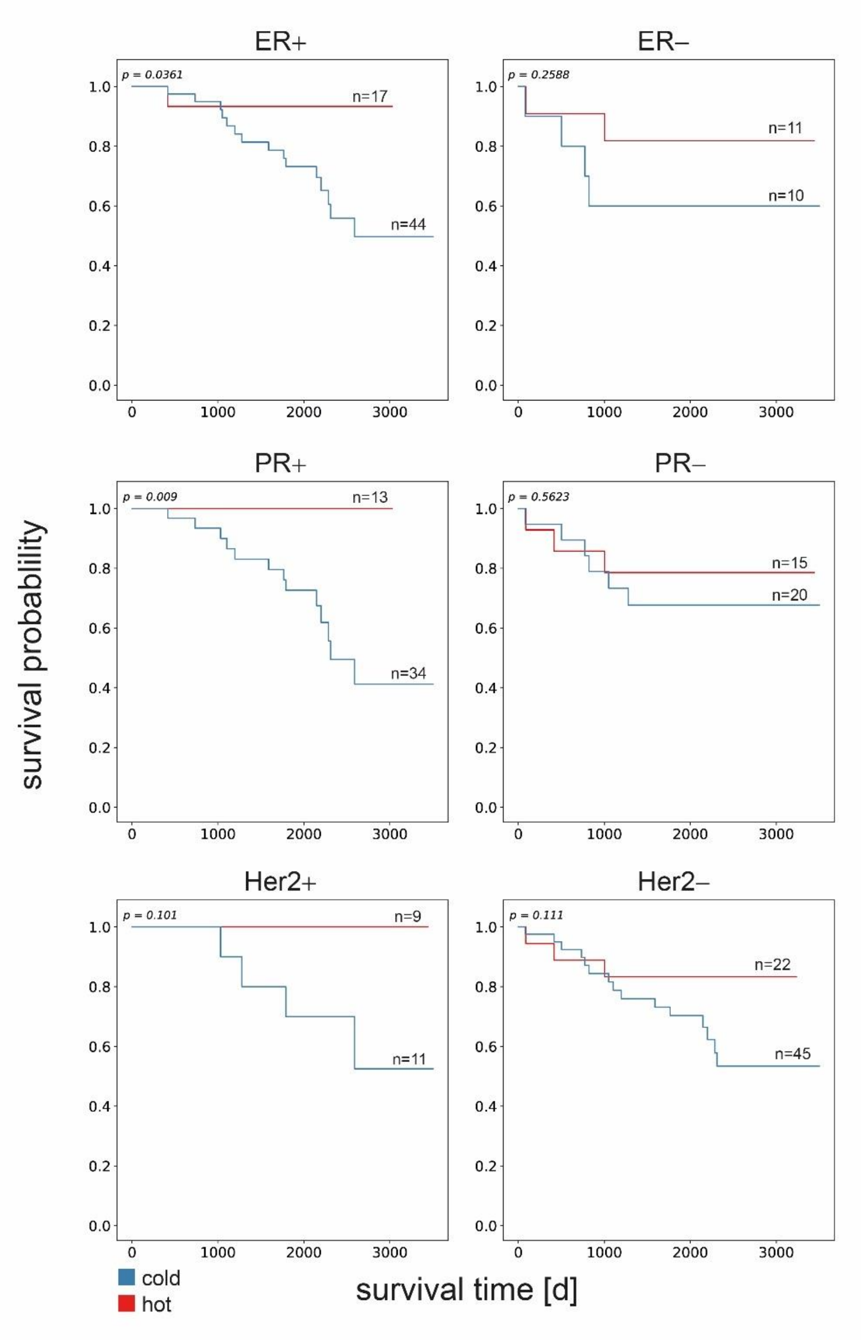

Supplementary Figure S5. Influence of infiltrating immune cells on event-free survival in hormone receptor and Her2 positive/negative tumors. Kaplan-Meier analysis of event-free survival between hot and cold carcinoma samples in ER, PR and Her2 positive and negative subgroups. A significant difference in EFS was found in ER+ and PR+ subgroup ($P<0.05$). Log-rank test.

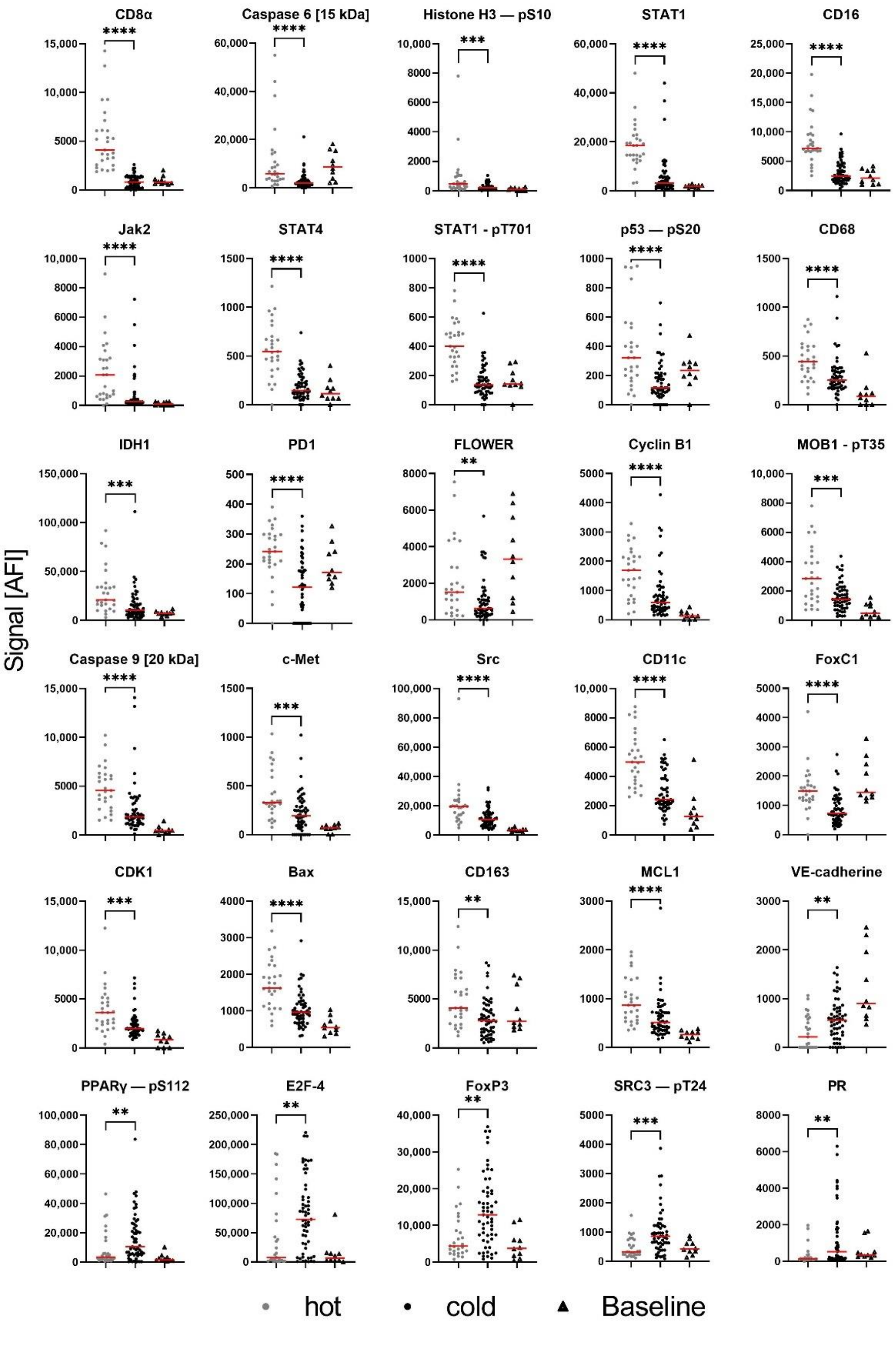

Supplementary Figure S6. Differences in protein expression between hot and cold tumors. Protein expression of 30 analytes for hot (n=27), cold (n=57) and baseline (n=10) subgroup which revealed significant differences in protein expression and a fold change of at least 2/3 between hot and cold samples. Mann-Whitney-U test, ****P<0.0001; ***P<0.001; **P<0.01.

Table S1. Correlation values for all measured immune cell markers. Spearman's correlation.

	CD4	CD25	CD56	CD163	CD11c	CD68	CD16	CD8a
**CD4**	1	-0.2	0.4	-0.4	0.1	0.2	0	0
**CD25**	-0.2	1	0.4	0.2	0.1	0.2	0.5	0.5
**CD56**	0.4	0.4	1	0	0.4	0.5	0.4	0.5
**CD163**	-0.4	0.2	0	1	0.5	0.4	0.3	0.4
**CD11c**	0.1	0.1	0.4	0.5	1	0.7	0.6	0.6
**CD68**	0.2	0.2	0.5	0.4	0.7	1	0.7	0.6
**CD16**	0	0.5	0.4	0.3	0.6	0.7	1	0.7
**CD8a**	0	0.5	0.5	0.4	0.6	0.6	0.7	1
